# Enhanced Anti-tumor of Pep-1 Modified Superparamagnetic Iron Oxide/PTX Loaded Polymer Nanoparticles

**DOI:** 10.3389/fphar.2018.01556

**Published:** 2019-01-22

**Authors:** Baoyan Wang, Weijun Wu, Hongjin Lu, Zhi Wang, Hongliang Xin

**Affiliations:** ^1^Nanjing Drum Tower Hospital, The Affiliated Hospital of Nanjing University Medical School, Nanjing, China; ^2^Department of Pharmaceutics, School of Pharmacy, Nanjing Medical University, Nanjing, China; ^3^Department of Pharmacy, The Second Affiliated Hospital of Nanjing Medical University, Nanjing, China

**Keywords:** tumor, drug delivery system, superparamagnetic iron oxide nanoparticle, interleukin-13 receptor α2, dual targeting nanocarrier

## Abstract

Superparamagnetic iron-oxide nanoparticle (SPION) has gained tremendous attention for drug delivery applications due to their unique properties. In this study, we developed a dual targeted delivery system with paclitaxel (PTX) and SPION co-loaded PLGA nanoparticles, modified with Pep-1 peptide (Pep-NP-SPION/PTX), to achieve magnetic targeting and active targeting for tumor treatment. SPION was synthesized by a co-precipitation method and was then encapsulated with PTX simultaneously into PLGA nanoparticles. After that, the non-complex was conjugated with Pep-1 through chemical modification. The resulting Pep-NP-SPION/PTX showed a spherical morphology and an average size of 100 nm. The enhancement cellular uptake of Pep-NP-SPION was demonstrated in *in vitro* through cell experiments. The IC_50_ value of Pep-NP-SPION/PTX and NP-SPION/PTX was determined to be 10.2 and 19.4 μg/mL, respectively. A biodistribution study showed that obvious higher accumulations of Pep-NP-SPION was observed in tumors, compared with that of non-targeting nanocomposites. Moreover, under the condition of a magnetic field, both NP-SPION and Pep-NP-SPION exhibited much higher tumor distribution. Furthermore, Pep-NP-SPION/PTX presented desirable *in vivo* anti-tumor effects based on active targeting and magnetic targeting characteristics. Altogether, Pep-NP-SPION/PTX can offer magnetic targeting and receptor mediated targeting to enhance the anti-tumor outcome.

## Introduction

Current tumor chemotherapy still faces a major problem with the of lack of selectivity of drugs on tumor cells, which leads to a narrow therapeutic index of most anti-tumor drugs (Wong et al., 2016). As a result, the achievement of an adequate therapeutic effect requires a high concentration of anti-tumor drugs, which in turn enhances the systematic toxicity. Thus, in order to reduce the toxicity of a chemotherapy drug at its minimum dose, specific nanoparticulate drug delivery systems, such as nanoparticle, liposome and polymeric micelle, could provide a non-invasive treatment strategy due to its passive targeting properties (Huang et al., 2016; Muralidharan et al., 2017; Roberts et al., 2017). However, these conventional drug delivery systems lack the capability of active targeting for tumor section.

Recently, superparamagnetic iron-oxide nanoparticles (SPION) consisting of cores made of iron oxides, have been considered to be attractive in cancer theranostic applications, since they can be delivered to the required tissue through an external magnetic field (Hachani et al., 2016). Due to the superparamagnetism, a high saturation field and a high field irreversibility, SPION has been widely used for various biological applications (Li et al., 2016). Moreover, SPIONs could lose their magnetization and become highly dispersed even after the removal of the magnetic field (Nagesh et al., 2016). However, biological application of SPION was limited because of the high surface hydrophobicity, making them susceptible to being ingested and eliminated by mononuclear phagocyte systems (MPS). In order to prolong the circulation time, it is necessary to modify the surface of SPION by amphiphilic copolymer coating to convert hydrophobic SPION into hydrophilic ones (Mirsadeghi et al., 2016; Silva et al., 2016).

Under an external magnetic field, SPION-encapsulated polymer nanoparticles could easily reach around the tumor section. In order to increase the active targeting of nanoparticles and uptake by tumor cells, receptor-mediated endocytosis could serve as a versatile targeting strategy *via* linking nanoparticles with multifunctional ligands to construct a potentially multiple targeting drug nanocarrier (Zhao et al., 2015; Rabiej et al., 2016; Shen et al., 2018). It is reported that various receptors are over-expressed on tumor cells, including folate receptor, neuropilin-1 receptor and transferrin receptor (Wang et al., 2011; Du et al., 2015; Song et al., 2015). Based on these receptors, drug delivery systems have been modified with corresponding targeting ligands and explored to deliver drugs through receptor-mediated endocytosis. The interleukin 13 receptor α2 (IL-13Rα2) is a subtype of the interleukin-13 receptor family, which is over-expressed on tumor cells (Mintz et al., 2002; Balyasnikova et al., 2012). It has been reported that IL-13Rα2, acting as a decoy receptor, has an intimate relationship with the progression of a tumor and can undergo internalization after binding to ligands (Kawakami et al., 2001). This property indicates that IL-13Rα2 could serve as a promising targeted moiety for anti-tumor drug delivery.

**FIGURE 1:**
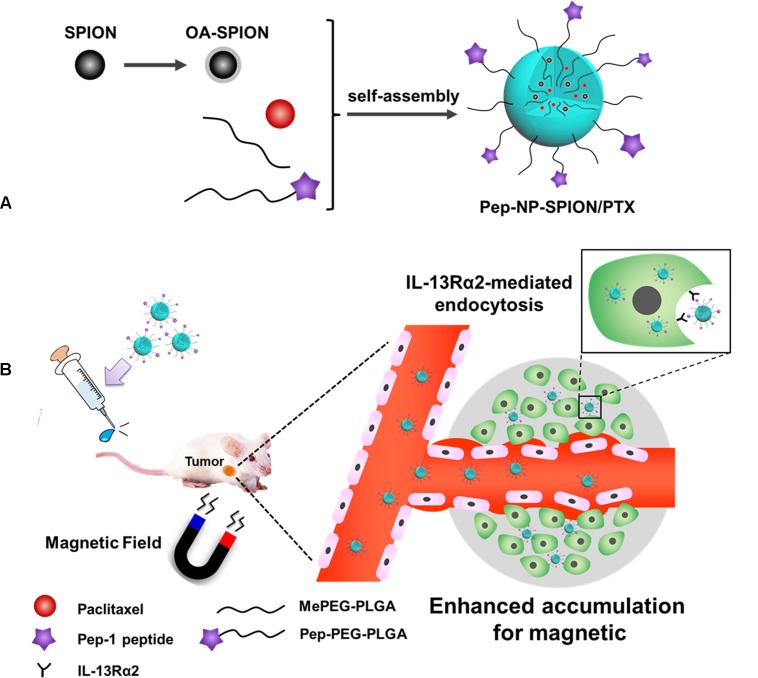
Schematic design of Pep-NP-SPION/PTX. The main components and preparation of the Pep-NP-SPION/PTX **(A)**. After intravenous injection, Pep-NP-SPION/PTX targets to tumor tissue *via* external magnetic field and then is internalized into tumor cells through IL-13Rα2 mediated endocytosis **(B)**.

Pep-1 peptide (CGEMGWVRC) that was screened by the phage display library, could bind to IL-13Rα2 with high affinity and specificity and could be exploited to target ligand to tumor cells (Pandya et al., 2012). In our previous study, we demonstrated that Pep-1 conjugated paclitaxel (PTX) loaded nanoparticles, could be internalized into tumor cells *via* IL-13Rα2 mediated endocytosis (Wang et al., 2014). However, the accumulation of the targeted drug delivery system in the tumor tissue was still rather low and could only be by enhanced penetration and retention (EPR) effects (Wilhelm et al., 2016). Therefore, we aimed at developing PTX and SPION co-loaded polymer nanoparticles with Pep-1 peptide modification as a dual targeting nanocarrier (designated as Pep-NP-SPION/PTX) for tumor treatment in this study. SPION was prepared using a co-precipitation method and loaded into PEG-PLGA polymer nanoparticles that were modified with Pep-1 peptide to form Pep-NP-SPION/PTX (Figure 1A). As showed in Figure 1B, after intravenous (i.v.) injection, Pep-NP-SPION/PTX was expected to accumulate at the tumor tissue in the presence of an external magnetic field and then be internalized into tumor cells through IL-13Rα2 mediated endocytosis, which would reduce the uptake of Pep-NP-SPION/PTX by the MPS and enhance the anti-tumor efficiency of PTX. These physical-chemical properties of the dual targeted nanocarrier were also systematically characterized. Furthermore, the *in vitro* biological targeted capability of Pep-NP-SPION/PTX was investigated. Finally, the *in vitro* and *in vivo* anti-tumor effect of Pep-NP-SPION/PTX was studied using a cell and subcutaneous xenograft tumor mice model, respectively.

## Materials and Methods

### Materials

Qleic acid, Iron(II) chloride, iron(III) chloride and ammonium hydroxide were purchased from Sinopharm Chemical Reagent Co., Ltd. (China). Methoxyl poly(ethylene glycol)-co-poly(D,L-lactic-co-glycolic acid) copolymer(MePEG-PLGA, 40 KDa) and Maleimidyl-poly(ethylene glycol)-co-poly(D,L-lactic- coglycolic acid) copolymer(Male-PEG-PLGA, 41.5 KDa) were purchased from Daigang Biomaterial Co., Ltd. (Jinan, China). PTX was purchased from Zelang Medical Technology Co., Ltd. (Nanjing, China). 3-(4, 5-dimethylthiazol-2-yl)-2, 5-diphenyltetrazolium bromide (MTT) was purchased from Beyotime Biotechnology Co., Ltd. (Nantong, China). Penicillin-streptomycin, RPMI 1640 medium, fetal bovine serum (FBS) and 0.25% (w/v) trypsin solution were obtained from Gibco BRL (Gaithersburg, MD, United States).

### Cell Line

The C6 cell line was obtained from the Institute of Biochemistry and Cell Biology, Shanghai Institutes for Biological Sciences, Chinese Academy of Sciences (Shanghai, China). The cell line was cultured in a RPMI 1640 medium, supplemented with 10% FBS, 1% penicillin and 100 mg/mL streptomycin sulfate. Cells were cultured in incubators maintained at 37°C with 5% CO_2_. All experiments were performed in the logarithmic phase of cell growth.

### Animals

Mice (male, 4–5 weeks, 20–25 g) were supplied by the Department of Experimental Animals, Nanjing Medical University (Nanjing, China) and maintained under standard a housing environment. All animal experiments were performed in accordance with protocols and evaluated and approved by the ethics committee of Nanjing Medical University.

### Synthesis of SPION Coated With Oleic Acid (OA-SPION)

Superparamagnetic iron-oxide nanoparticle was prepared by co-precipitating ferrous and ferric salts in an alkaline medium (Schleich et al., 2013). Briefly, 0.10 g of FeCl_2_⋅4H_2_O and 0.27 g of FeCl_3_⋅6H_2_O were added into 50 mL PEG600, which was placed under a vacuum overnight at 40°C to degas. The mixture was then heated to 125°C under nitrogen protection. Then, 2 mL ammonium hydroxide was added, and the mixture was reheated to 125°C. 5 min later, 20 mL of oleic acid was added into the solution at 125°C and the mixture was kept at this temperature for another 30 min. Finally, the black precipitate was separated by an external magnet and washed three times with absolute ethanol and ultrapure water to remove the unreacted reagent, respectively. Dry SPION coated with oleic acid was prepared through freeze-drying for further use.

### Preparation of Pep-NP-SPION/PTX

Paclitaxel and SPION loaded PLGA-based nanoparticles were prepared *via* an emulsion/solvent evaporation method (Wilhelm et al., 2016). Firstly, the conjugation of Pep-1 peptide to PEG-PLGA copolymer *via* the reaction of maleimide with thiol group was carried out as previously reported (Wilhelm et al., 2016). Then, 19 mg MePEG-PLGA and 2 mg Pep-PEG-PLGA polymer were dissolved in 1 mL dichloromethane (DCM) containing PTX (1 mg/mL) and OA-SPION (Fe concentration: 4 mg/mL). Then the mixture was added to a 2 mL 1% (w/v) sodium cholate aqueous solution, which was finally sonicated using a probe sonicator (Xin Zhi Biotechnology Co., Ltd., China) for 5 min at 190 W output. The suspension was added drop-wisely into 10 mL 1% (w/v) sodium cholate solution under moderate stirring. A rotary evaporator was used to evaporate the redundant DCM at 40°C. The solutions were centrifuged at 13500 *g* for 40 min to remove the excess excipient. After being washed three times by water, the Pep-NP-SPION/PTX was collected and re-dispersed in PBS for further use.

### Characterization of Pep-NP-SPION/PTX

#### Morphology, Particle Size and Zeta Potential of Pep-NP-SPION/PTX

The morphology of Pep-NP-SPION/PTX was characterized by the transmission electron microscope (TEM, Philips CM 100) operating at a voltage of 100 kV. The particle size and zeta potential of nanoparticles were assessed by dynamic light scattering (DLS, Zs90, Malvern, United Kingdom).

#### Determination of SPION Loading Content

The Fe content was measured using the phenanthroline spectrophotometric method. Lyophilized nanoparticles were dissolved in DCM, and then dried under nitrogen. The residue was dissolved with 10% (v/v) HCl solution. The yellow solution was combined with aqueous solutions of hydroxylamine hydrochloride (10%, w/v) and sodium acetate buffer solution (pH = 5, 1 mol/L). Then 0.1% phenanthroline solution was added. After 0.5 h, the absorbance at 510 nm was measured and the Fe content was determined based on a comparison of a standard curve.

#### Encapsulation Efficiency and Loading Capacity of PTX

The amount of PTX was measured by high performance liquid chromatography (HPLC) with UV detection at 227 nm (LC-10AT, SHIMADZU, Japan). The mobile phase consisted of acetonitrile and water (47:53, v/v) with a gradient elution pumped at a flow rate of 1.0 mL/min and the column was maintained at 30°C. The calibration curve was linear in the range of 0.1–100 μg/mL with a correlation coefficient of *R*^2^ = 0.9998.

To determine the encapsulation efficiency (EE%) and loading capacity (LC%) of Pep-NP-SPION/PTX, the lyophilized samples were dissolved in DCM, and then dried under nitrogen. The residue was dissolved in mobile phase solution and the concentration of PTX was analyzed by HPLC as described. The EE% and LC% were calculated as indicated below (*n* = 3).

$$\mathrm{EE}\%=\frac{\mathrm{Amount of PTX in the nanoparticles}}{\mathrm{Total amount of PTX added}}\times 100\%$$

$$\mathrm{LC}\%=\frac{\mathrm{Amount of PTX in the nanoparticles}}{\mathrm{Nanoparticles weight}}\times 100\%$$

### *In vitro* Release

The *in vitro* release kinetics of PTX from Pep-NP-SPION/PTX was measured by a standard dialysis method in PBS (0.04 M, pH 7.4) or HAc-NaAc buffer (0.04 M, pH 5.0) containing 0.1% (w/v) Tween-80 at 37°C. Briefly, nanoparticles were suspended in 1 mL of mediator solution and placed in a dialysis bag (MWCO 3000). The dialysis bag was immersed in 30 mL of the release medium which was shaken at 37°C. At predetermined time intervals, a portion of 0.3 mL dialysate was harvested, and the same volume of fresh mediator solution was added. The PTX concentration of samples was determined by HPLC as described above.

### *In vitro* Evaluation

#### *In vitro* Cellular Uptake

C6 cells were seeded into a 24-well plate at the density of 1 × 10^5^ cells per well. After 24 h incubation, the cells were incubated with NP-SPION and Pep-NP-SPION at different concentrations (Fe concentration: 25 and 50 μg/mL) at 37°C for 1 h, respectively. After that, the cells were washed three times with cold PBS and fixed with 4% formaldehyde for 10 min. Then, these cells were stained with Pearls’ reagent (2% potassium ferrocyanide/6% HCl: 1/1) for 30 min. Finally, after washing by PBS for three times, the uptake of nanoparticles in the C6 cells was observed using a microscope.

#### *In vitro* Cytotoxicity

An MTT assay was used to evaluate the cell cytotoxicity of Pep-NP-SPION/PTX. C6 cells were seeded into 96-well plates at the density of 5000 cells/well and incubated at 37°C in a 5% CO_2_ atmosphere, and then incubated with Taxol^®^, NP-SPION/PTX and Pep-NP-SPION/PTX at different PTX concentration (1, 5, 10, and 20 μg/mL) after 24 h, respectively. All the concentrations of Fe used were based on the corresponding PTX concentrations (Fe concentrations: 3.9, 19.6, 39.2, and 78.4 μg/mL). After 48 h, the culture medium was discarded and 20 μL MTT solution was added into each well and incubated for additional 4 h. Then the unreacted dye was removed and 200 μL of DMSO was added to each well. Finally, the optical density was measured by a microplate reader at wavelength of 490 nm.

### *In vivo* Evaluation

#### Biodistribution of Pep-NP-SPION/PTX in Tumor Tissue

C6 cells (3 × 10^6^ cells suspended in 100 μL PBS) were injected into the armpit of the right anterior limbs of mice subcutaneously. When the tumor volume reached about 300 cm^3^, the mice were divided into four groups randomly, then intravenously administrated with NP-SPION/PTX and Pep-NP-SPION/PTX (Fe dose of 15 mg/kg), respectively. The influence of the external magnetic field was also evaluated. 2 h after injection, the mice were sacrificed, and the tumor tissues were harvested and fixed in 4% paraformaldehyde. After paraffin embedding, the tumors were cut into 5 μm and stained with Prussian blue staining.

#### *In vivo* Anti-tumor Efficacy

*In vivo* tumor growth inhibition was carried out to evaluate the anti-tumor efficacy of Pep-NP-SPION/PTX. Subcutaneous xenograft tumor mice model was established as described above. With the tumor volume reached about 100 mm^3^, the mice were divided into six groups randomly, and then intravenously administrated with saline, Taxol^®^, NP-SPION/PTX and Pep-NP-SPION/PTX (PTX dose: 4.5 mg/kg, Fe dose: 15.6 mg/kg), respectively. The influence of external magnetic field was also evaluated. The formulation was given every other day for four injections. 15 days later, the mice were sacrificed with tumor collection and tumor volume was calculated (the formula: π/6 × larger diameter × smaller diameter^2^).

## Results

### Synthesis of SPION Coated With Oleic Acid

**FIGURE 2:**
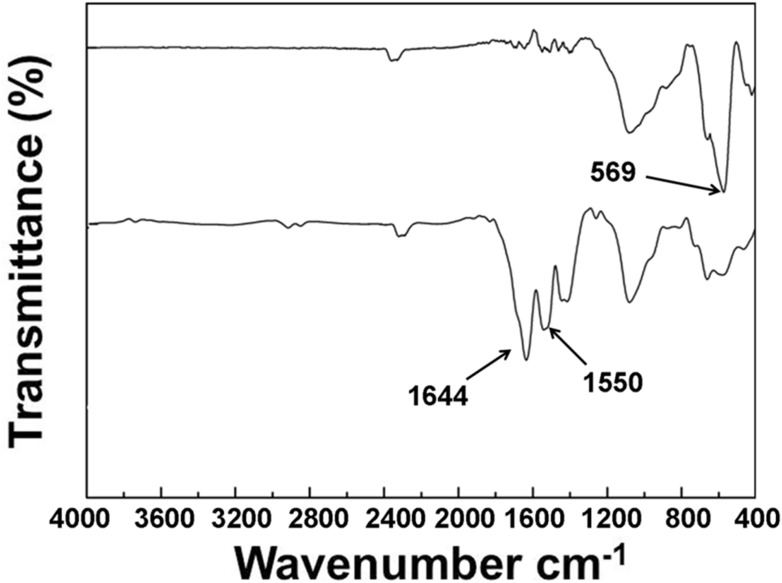
FTIR spectra of SPION and OA-SPION.

In order to improve the hydrophobicity and dispersibility of SPION, their surfaces were conjugated by oleic acid through physical adsorption. The FTIR spectra of SPION and OA-SPION are shown in Figure 2. The absorption peak at 569 cm^−1^ was the characteristic absorption peak of Fe-O bond. The spectrum of OA-SPION showed a sharp absorption peak at 1644 cm^−1^, which was attributed to the C = O stretching vibrational absorption of oleic acid. However, the characteristic vibrational absorptions were not present in the spectrum of SPION. These results demonstrated the successful modification of oleic acid on the surface of SPION.

### Characterization of Pep-NP-SPION/PTX

**FIGURE 3:**
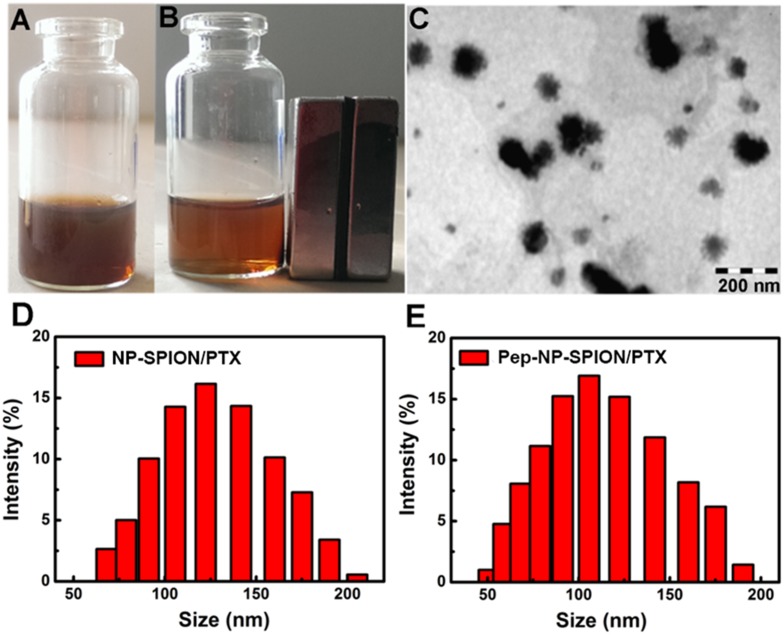
Characterization of Pep-NP-SPION/PTX. Pep-NP-SPION/PTX solution **(A)** or absorption under external magnetic field **(B)**; TEM image of Pep-NP-SPION/PTX **(C)** and size distribution of NP-SPION/PTX **(D)** and Pep-NP-SPION/PTX **(E)**.

Pep-NP-SPION/PTX was prepared *via* an emulsion/solvent evaporation method. As shown in Figure 3, Pep-NP-SPION/PTX was well-dispersed in water and showed a brown color before magnetic separation. When exposed to a magnetic field, the solution become more and more colorless and the nanoparticles were collected near the magnet. Therefore, the magnetic property of SPION was not affected by the encapsulation of PEG-PLGA.

The morphology and size of Pep-NP-SIPION/PTX was characterized by TEM and DLS, respectively. A TEM photograph showed that Pep-NP-SPION/PTX was uniformly spherical and of regular shape with a narrow size distribution. As shown in Table 1, the particle size of NP-SPION/PTX and Pep-NP-SPION/PTX were 129.8 ± 1.2 nm and 101.6 ± 1.0 nm, respectively. The particle size difference between NP-SPION/PTX and Pep-NP-SPION/PTX might be due to the modification of hydrophilic Pep-1 peptides, which enhanced the hydrophilicity of polymer nanoparticles. The zeta potential of NP-SPION/PTX and Pep-NP-SPION/PTX were below −20 mV.

**Table 1:** Characterization of NP-SPION/PTX and Pep-NP-SPION/PTX.

	NP-SPION/PTX	Pep-NP-SPION/PTX
Particle size (nm)	129.8 ± 1.2	101.6 ± 1.0
Zeta potential (mV)	−21.9 ± 0.5	−21.2 ± 0.5
Encapsulation efficiency (EE%)	66.85 ± 1.84	81.30 ± 3.50
Loading capacity (LC%)	3.22 ± 0.86	3.54 ± 0.14
Iron loading (mg/100 mg polymer)	12.41 ± 2.75	13.53 ± 3.38

The PTX LC% and EE% of Pep-NP-SPION/PTX were 3.54 ± 0.14% and 81.30 ± 3.50%, respectively, compared with 3.22 ± 0.86% and 66.85 ± 1.84% for NP-SPION/PTX. The change of encapsulation efficiency and loading capacity might be attributed to the modification of polymer materials with Pep-1 peptide. The iron loading of NP-SPION/PTX and Pep-NP-SPION/PTX was 12.41 ± 2.75 mg and 13.53 ± 3.38 mg/100 mg polymer, respectively.

### *In vitro* Drug Release

**FIGURE 4:**
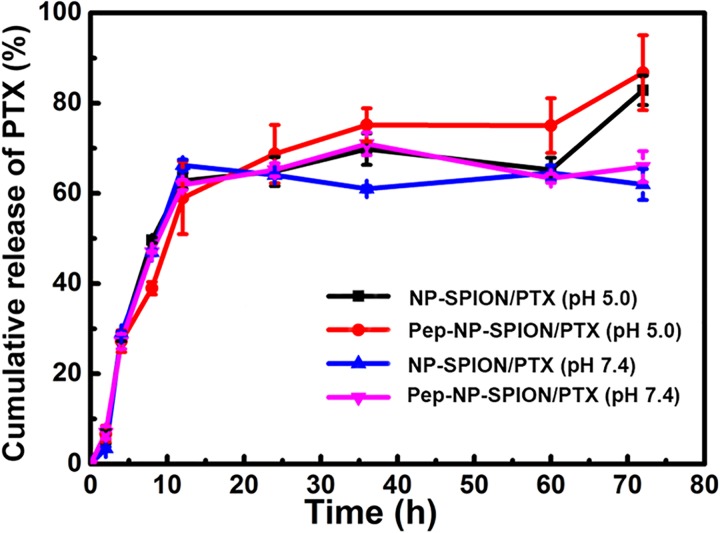
PTX release profiles from NP-SPION/PTX and Pep-NP-SPION/PTX in HAc-NaAc (pH 5.0) and PBS (pH 7.4) buffer solution containing 0.1% (w/v) Tween-80.

The release profiles of PTX-loaded magnetic nanoparticles under different pH conditions are presented in Figure 4. Tween-80 was used to increase the solubility of PTX in a buffer solution and to avoid the binding of PTX to the nanoparticle surface. Herein, the drug release behavior was studied under a simulated physiological environment (pH 7.4) and an acidic condition (pH 5.0) because of the lower pH value in tumor tissues, due to excess lactic acid produced by hypoxia and acidic intracellular organelles (Xu et al., 2012).

Paclitaxel-loaded magnetic nanoparticles all exhibited an initial burst release profile with total PTX releases of about 60% before 12 h. After 72 h, NP-SPION/PTX and Pep-NP-SPION/PTX showed a total drug release of 63.4 and 65.8% of PTX at pH 7.4, respectively, while 82.3 and 87.1% of the PTX in PBS (pH 5.0), respectively. These results suggested that solution pH could affect the release pattern of PTX from nanoparticles. The pH responsive release of the dual targeted nanoparticles may be beneficial for enhancing the anti-tumor efficacy of the PTX in the acidic tumor microenvironment.

### *In vitro* Cell Experiment

#### *In vitro* Cellular Uptake

**FIGURE 5:**
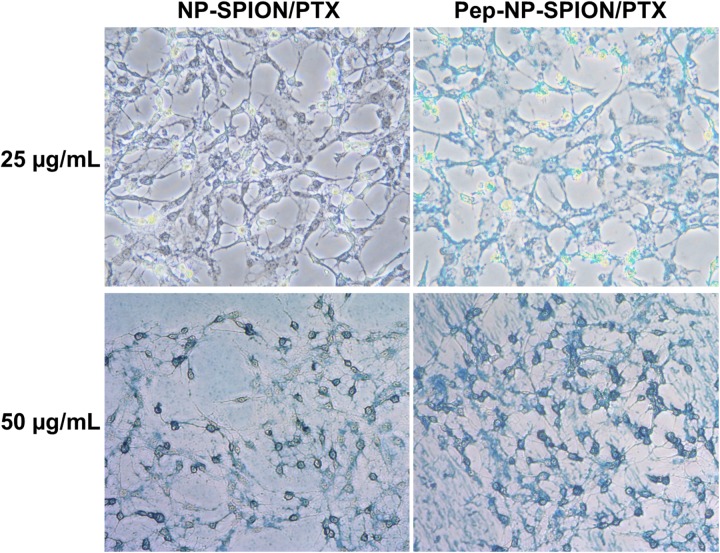
Prussian blue staining images of C6 cells after 1 h incubation with NP-SPION/PTX and Pep-NP-SPION/PTX with Fe concentration at 25 and 50 μg/mL. Original magnification: 200×.

It has been reported that IL-13Rα2 was upregulated in C6 cell line (Daines et al., 2006), so the C6 cell was used as the cell model to investigate the *in vitro* uptake of Pep-NP-SPION in this study. The cellular uptake of Pep-NP-SPION was studied quantitatively by Prussian blue staining. The number of blue granules within C6 cells was related to the nanoparticle concentration. As shown in Figure 5, the blue granules intensity was increased with the increase of iron concentrations ranging from 25 to 50 μg/mL after 1 h incubation, which indicated that the cellular uptake of Pep-NP-SPION exhibited a concentration-dependent mode. Moreover, the cellular uptake of Pep-NP-SPION was obviously higher than that of NP-SPION. These results implied that the modification of Pep-1 peptide could enhance the cellular uptake of nanoparticles through receptor-mediated endocytosis.

#### *In vitro* Cytotoxicity

**FIGURE 6:**
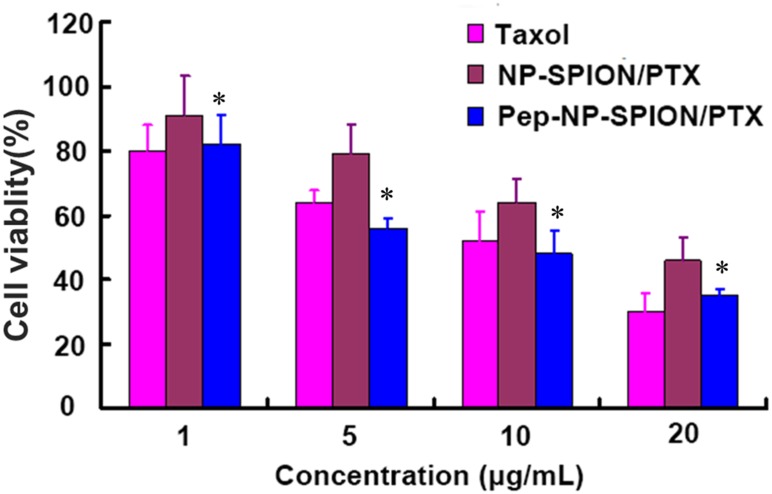
*In vitro* cytotoxicity of Taxol^®^, NP-SPION/PTX and Pep-NP-SPION/PTX at various concentrations at 48 h. Data represented mean ± SD (*n* = 3). ^∗^*p* < 0.05 significantly lower than that of NP-PTX.

The *in vitro* cell viability of NP-SPION/PTX and Pep-NP-SPION/PTX was evaluated by MTT assay. The cytotoxicity of various PTX formulations exhibited a concentration-dependent pattern with the increase of PTX concentration ranging from 1 to 20 μg/mL. Different degrees of cytotoxicity were found in all the PTX formulations. As shown in Figure 6, the cell viability of Pep-NP-SPION/PTX was even lower than 50% at PTX concentration of 10 μg/ml. The IC_50_ value of Pep-NP-SPION/PTX and NP-SPION/PTX is 10.2 and 19.4 μg/mL, respectively.

### Biodistribution of Pep-NP-SPION/PTX in Tumor Tissue

**FIGURE 7:**
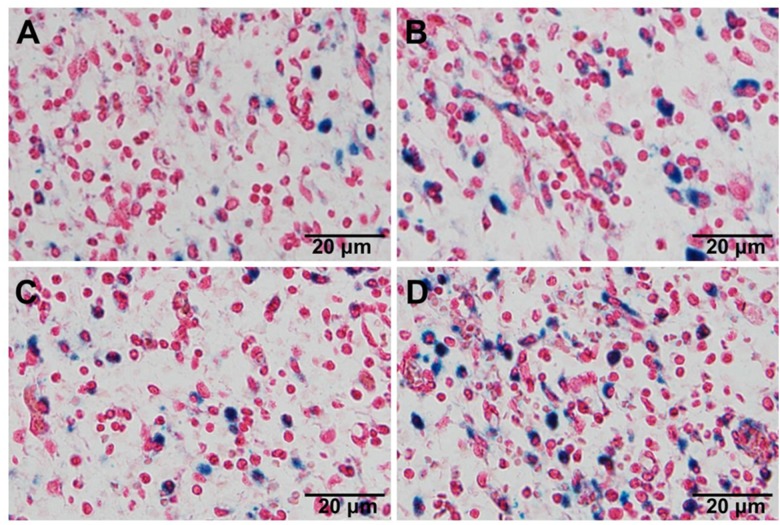
The distribution of nanoparticles in tumor section visualized by Prussian blue staining. Histological sections were extracted at 2 h after intravenous injection of NP-SPION/PTX **(A)**, NP-SPION/PTX with magnetic field **(B)**, Pep-NP-SPION/PTX **(C)**, and Pep- NP-SPION/PTX with external magnetic field **(D)**.

The *in vivo* tumor targeting capability of magnetic nanoparticles was studied qualitatively by Prussian blue staining. Iron oxide was indicated as blue and cell nucleus as red. As shown in Figure 7, in the presence of an external magnetic field, both NP-SPION/PTX and Pep-NP-SPION/PTX exhibited much higher tumor distribution than those without magnetic field intervention, which indicated that the constructed nanoparticles had an attractive magnetic targeting character for improving tumor distribution. Moreover, compared with the NP-SPION/PTX group, Pep-NP-SPION/PTX showed an obvious higher distribution no matter whether an external magnetic field was added, which revealed that the modification of Pep-1 peptide could accelerate the accumulation of nanoparticles into the tumor section *via* IL-13Rα2 mediated endocytosis. Altogether, based on the active targeting and magnetic targeting, Pep-NP-SPION/PTX can be used as a potential dual targeting nanocarrier for the treatment of tumors.

### *In vivo* Anti-tumor Efficacy

**FIGURE 8:**
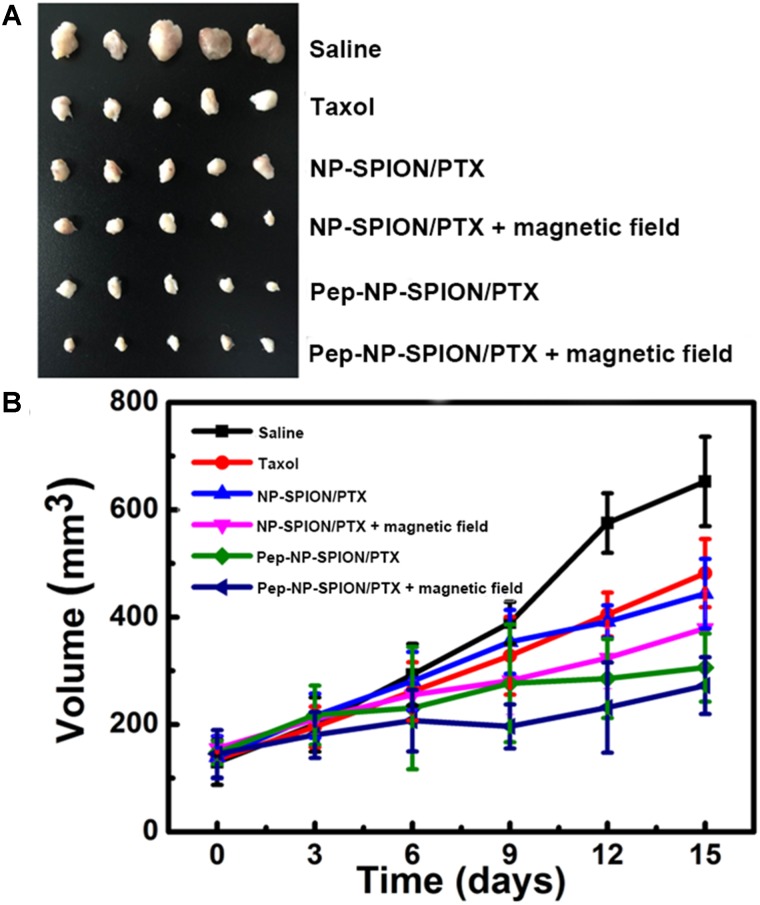
Anti-tumor efficacy of Taxol^®^, NP-SPION/PTX and Pep-NP-SPION/PTX on tumor xenograft mice. Tumor xenografts alignment of each group taken out from the sacrificed mice at the end point **(A)** and tumor growth volume vs. time **(B)**. (*n* = 5).

In this study, mice bearing subcutaneous tumor xenograft were used to evaluate the *in vivo* anti-tumor efficacy of Pep-NP-SPION/PTX. The mice were intravenously injected with Taxol, NP-SPION/PTX and Pep-NP-SPION/PTX (PTX dose: 4.5 mg/kg, Fe dose: 15.6 mg/kg) every other day for four consecutive administrations with tumor sizes recorded every 3 days, respectively. As shown in Figure 8, the tumor size of the saline group was obviously all larger than that of the PTX formulations. In the initial days, there was no significant difference in tumor size among these groups. At the experimental terminal, the tumor size of all PTX formulations followed the order: Pep-NP-SPION/PTX with magnetic field < Pep-NP-SPION/PTX < NP-SPION/PTX with magnetic field < NP-SPION/PTX < Taxol < Saline. These results showed that the modification of Pep-1 peptide could improve the anti-tumor efficacy of nanoparticles through IL-13Rα2-mediated endocytosis, consistent with our previous study. Moreover, the encapsulation of SPION endowed nanoparticles with the magnetic targeting property and enhanced the anti-tumor efficacy with the magnetic field. Together, Pep-NP-SPION/PTX constructed in this study could offer a potential magnetic targeting and receptor mediated targeting to enhance the anti-tumor efficacy for tumor treatment.

## Conclusion

In this study, we successfully constructed PTX and SPION co-loaded polymer nanoparticles with Pep-1 peptide modification as a dual targeted nanocarrier for tumor treatment. The cellular uptake of Pep-NP-SPION/PTX showed a concentration-dependent manner and significantly enhanced than that of unmodified NP-SPION/PTX. Pep-NP-SPION/PTX exhibited cytotoxicity comparable to Taxol at the PTX concentrations ranging from 1 to 20 μg/mL *in vitro* cell experiments. Furthermore, Pep-NP-SPION/PTX showed a satisfactory tumor accumulation and the magnetic field significantly enhanced the bio-distribution of nanoparticles in the tumor section. More importantly, the *in vivo* anti-tumor efficacy showed that Pep-NP-SPION/PTX exhibited desirable anti-tumor efficacy and the magnetic field could also enhance the anti-tumor efficacy. Altogether, these results indicate that the modification of Pep-1 peptide could enhance the active targeting property through receptor-mediated endocytosis and the encapsulation of SPION which obviously improves the physical targeting property of nanoparticles under an external magnetic field. The physical magnetic targeting and IL-13Rα2 mediated active targeting characteristics could synergistically increase the targeted efficiency for a tumor. Therefore, Pep-NP-SPION/PTX could serve as a potential dual targeting nanocarrier for tumor therapy.

## Author Contributions

HX designed the experiments. BW and WW performed the experiments. BW and HX wrote the main manuscript. HL and ZW prepared the figures and tables. All authors reviewed the manuscript.

## Conflict of Interest Statement

The authors declare that the research was conducted in the absence of any commercial or financial relationships that could be construed as a potential conflict of interest.
